# Introduction to *Applied Biosafety*’s Second Special Issue on Synthetic Genomics

**DOI:** 10.1089/apb.2024.0015

**Published:** 2024-09-18

**Authors:** Sarah R. Carter, Rocco Casagrande, Scott J. Patlovich, David R. Gillum

**Affiliations:** ^1^ Science Policy Consulting LLC, Arlington, Virginia, USA.; ^2^ Gryphon Scientific, Takoma Park, Maryland, USA.; ^3^ The University of Texas Health Science Center at Houston, Houston, Texas, USA.; ^4^ Compliance and Research Administration, University of Nevada, Reno, Reno, Nevada, USA.

As the capabilities of synthetic biology continue to expand, bringing both monumental scientific benefits and unprecedented challenges, the dialogue surrounding biosafety and biosecurity becomes increasingly urgent. This second Special Issue, which continues to feature biosafety and biosecurity considerations for synthetic genomics, explores the evolving landscape of synthetic genomics through a lens that balances innovation with the imperative for security. The manuscripts compiled here provide critical insights into emerging biosecurity concerns, the development of new safety protocols, and the ethical considerations necessary in this rapidly advancing field.

Millett’s discussion outlines biosecurity responsibilities when reporting on the vulnerabilities in nucleic acid synthesis screening. As more players outside of industry recognize the importance of biosecurity in this industry, elaborating standards for red-teaming helps ensure that these activities don’t exacerbate existing vulnerabilities or undermine the industry they are trying to support.

Berger et al. contribute an essential perspective on the need for dynamic biosecurity frameworks that can adapt to the rapid pace of innovation in transdisciplinary biotechnologies. The authors argue for revised risk assessment methodologies that can keep pace with the enhanced capabilities provided by synthetic and engineering biology, thus ensuring that biosecurity measures are both current and comprehensive.

The prototype DNA synthesis screening test dataset developed by Wheeler et al. exemplifies a practical approach to enhancing biosecurity. By providing a method for benchmarking the performance of various DNA screening methods used by synthesis companies, this work contributes to efforts to compare these methods. This type of work is particularly urgent and relevant as the National Institute of Standards and Technology aims to develop standards for DNA synthesis screening as directed by the October 2023 Executive Order on the “Safe, Secure, and Trustworthy Development and Use of Artificial Intelligence.”

Shifting the focus of DNA synthesis screening to the functional attributes of sequences, as discussed by Godbold and Scholz, illustrates a more expansive approach to biosecurity. By identifying “Sequences of Concern” that contribute to pathogenicity or toxicity, this strategy enhances our ability to preemptively address a wider range of biothreats and incorporates a broader understanding of risk.

The work by Gemler et al. on the impact of ambiguities about the types of synthetic nucleic acids that should be considered for additional oversight raises crucial considerations about how clarity and precision in oversight frameworks can directly affect the efficacy of biosecurity measures. This discussion is particularly timely, given the increasing complexity of synthetic biology applications and their potential dual-use concerns.

Rose et al. address several pressing issues for policymakers, including the challenges of navigating customer and sequence screening within the tangled web of domestic and international oversight regimes. Their insights into the practicalities of ensuring adherence to these practices in both commercial and benchtop contexts are essential for developing coherent and effective policies.

Lastly, Adam and McArthur IV’s examination of “substitution attacks” within DNA synthesis technology highlights emerging cyberbiosecurity threats. Their call for increased vigilance and innovation in safeguarding synthesized genetic materials reflects the broader concerns about cybersecurity in biotechnology.

This second Special Issue not only maps the current landscape of biosafety and biosecurity challenges in synthetic genomics but also serves as a call to action. It emphasizes the need for continuous dialogue among scientists, safety professionals, policymakers, and the public to navigate the complexities of synthetic biology. Public concerns about the risks associated with biotechnology advances are legitimate and must be addressed transparently and effectively. Through comprehensive research, thoughtful policy development, and engaged ethical discussion, a balance can be achieved, ensuring that the benefits of synthetic biology are realized while safeguarding public health and environmental integrity.

